# Multicentre clinicopathological study of adenoid cystic carcinoma: A report of 296 cases

**DOI:** 10.1002/cam4.3707

**Published:** 2021-01-15

**Authors:** Zheng Huang, Juan Pan, Jiaorong Chen, Shidi Wu, Ting Wu, Haihua Ye, Hongfeng Zhang, Xiu Nie, Changzheng Huang

**Affiliations:** ^1^ Department of Pathology Central Hospital of Wuhan Tongji Medical College Huazhong University of Science and Technology Wuhan China; ^2^ Department of Pathology Union Hospital Tongji Medical College Huazhong University of Science and Technology Wuhan China; ^3^ Department of Anatomy and Histology & Embryology Basic Medical College Hubei University of Traditional Chinese Medicine Wuhan China; ^4^ Department of Dermatology Union Hospital Tongji Medical College Huazhong University of Science and Technology Wuhan China

**Keywords:** adenoid cystic carcinoma, clinicopathological analysis, metastasis, S100

## Abstract

**Aims:**

Adenoid cystic carcinoma (ACC) is a distinctive tumour. Limited studies involving a large population have reported multicentre systematic analyses of the clinical, pathological and immunohistochemical (IHC) features of ACC as well as the potential role of IHC markers in the prognosis of ACC.

**Methods and Results:**

The clinical, histopathological and IHC data of 296 cases obtained from two tertiary hospitals were analysed. The age at onset ranged from 12 to 87 years with a median age of 52 years. The male‐to‐female ratio was 1:1.3. Patients with ACC arising from the lacrimal gland were younger than those with tumours arising from other sites. Patients with tumours in the extra auditory canal and nasopharynx were older than those with tumours in other locations. Histopathologically, solid type ACC was the most frequent in the nasal cavity and paranasal sinus (6/51) group. Tumours arising from the oral cavity most commonly showed perineural invasion (10/60) and margin positivity (11/60). IHC analyses showed that CK8/18, CK7, CK14, epithelial membrane antigen and CD117 were expressed in 35/35 (100%), 87/88 (98.8%), 26/27 (96.2%), 42/43 (97.6%) and 113/120 (94.1%) patients, respectively. CK5/6, P63, smooth muscle actin, calponin and S100 were positively expressed in 73/73 (100%), 111/124 (89.5%), 38/43 (88.3%), 41/50 (82.0%) and 61/92 (66.3%) cases, respectively. S100 proteins were expressed in 54 (54/77) primary cases and two (2/9) metastatic cases (*p* = 0.013).

**Conclusions:**

ACC is a distinctive tumour that mainly affects middle‐aged and elderly individuals, with a mild female predominance. Loss of expression of S100 proteins may be a poor prognostic factor associated with metastasis.

## Graphical abstract

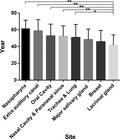

## 1 INTRODUCTION

Adenoid cystic carcinoma (ACC) is a distinctive tumour with a slight female predominance in terms of incidence.^1^ ACC can originate in any part of the body but is most commonly found in the salivary glands. The tumour can also be found outside the head and neck region, including the breast, skin, vulva and prostate. The primary site of cancer determines the prognosis.^2^

ACC is characterised by a paradoxical behaviour. The tumour is slow growing and indolent but is considered highly malignant in the long‐term. Although the 5‐year survival rate (approximately 70%) is relatively high, a low 15‐year survival rate (approximately 40%) has been reported.^3^

ACC is characterised as a tumour with bidirectional differentiation of myoepithelial and epithelial cells.^4^ Epithelial cells display CK7, CD117, epithelial membrane antigen (EMA), CK14, CK8/18 and CAM5.2, while myoepithelial cells express CK5/6, S100, P63, smooth muscle actin (SMA) and calponin.^4^, ^5^ S100 proteins, a group of calcium‐binding proteins, were initially considered unique to the nervous system; however, they are also expressed in other tissues/cells, such as myoepithelial cells (seen in ACC),^4^, ^5^ cartilage and fat.^6^ No studies have been reported on the role of S100 expression in the metastasis of ACC.

ACC is subdivided into three histological groups. The classical pattern is characterised by tumour cells arranged in a cribriform pattern. The tubular pattern is characterised by glands with a single lumen. The solid pattern is associated with poor prognosis.^7^ This pattern is characterised by predominantly epithelial differentiation with high‐grade features and is associated with a higher proliferation rate and a more aggressive nature. The poor prognosis associated with the solid pattern (compared to that with the other two subtypes) implies that the lack of myoepithelial cell differentiation is a predictor of poor disease outcomes.^4^

A previous study showed that ACC with high‐grade transformation could be histologically discriminated from traditional ACC based on a larger and more irregular nucleus, an increased mitotic count and the loss of bidirectional epithelial‐myoepithelial differentiation.^7^

Studies have reported that ACC expresses *myeloblastosis oncogene* (*MYB*), *NFIB* and/or *MYBL1* (at least one is expressed).^8^, ^9^, ^10^ *MYB* translocations were found in approximately 75% cases of ACC, making it the most common recurrent intrinsic genetic mutation. In addition, *MYB*‐*like 1* drives the MYB‐like expression pattern in ACC.^8^, ^9^, ^10^

Previous clinicopathological studies on ACC have focused on the head and neck region without differentiating anatomic sites or mixed tumour types with respect to one sub‐site. The current study is an inclusive study of the clinicopathological and immunohistochemical (IHC) results of 296 ACC cases focused on evaluating the diagnostic value and prognostic value of IHC markers in ACC.

## 2 MATERIALS AND METHODS

Data of 296 cases of ACC confirmed by haematoxylin‐eosin (HE) and IHC staining were collected from two tertiary hospitals, Wuhan Union Hospital and Wuhan Central Hospital, Tongji Medical College, Huazhong University of Science and Technology, Hubei province, China, from January 2012 to September 2019, following authorisation from both Institutional Ethics Committees or Review Boards. Sex, age, age at diagnosis, anatomic site, symptom presentation, duration of the disease, tumour size, histological type, margin status, perineural invasion, vascular invasion, lymph node (LN) stage, high‐grade transformation and IHC results were analysed for all cases. In cases in which the tumour size could not be determined or the primary site of the tumour was the nasal cavity and paranasal sinuses (the specimen was presented in a fragmented fashion and showed mixed site involvement), reports of imaging studies, such as computed tomography and magnetic resonance imaging studies, were used.

Solid type ACC was defined when the solid pattern accounted for over 30% of the tumour; otherwise, the tumour was diagnosed to be of the tubular/cribriform type.

All samples were routinely sectioned into 4‐μm thick slices for HE and IHC staining (standardised EnVision method). For IHC, Autostainer Link 48 platform (DAKO, Glostrup, Denmark) was used. Ready‐to‐use primary antibodies were purchased from DAKO (Glostrup, Denmark) or MXB (Fujian P.R. China). Details of the clones are listed in Table 1. Tris‐EDTA solution was routinely used for antigen retrieval.

**TABLE 1:** Detailed information of the primary antibodies used for analyses

Antibody	Clone (manufacturer)	Antibody	Clone (manufacturer)
Pan‐CK	AE1/AE3 (MXB)	CK19	RCK108 (DAKO); MX054 (MXB)
CK7	OV‐TL 12/30 (DAKO)	CD56	123C3 (DAKO)
EMA	E29 (DAKO)	P40	Multiclonal
CD117	Multiclonal (DAKO)	Bcl−2	124 (DAKO); MX022 (MXB)
CK8/18	EP17/EP30 (DAKO); 5D3 (MXB)	SMMHC	SMMS−1 (NOVODIAX)
CAM5.2	CAM5.2 (MXB)	CK20	Ks20.8 (DAKO); MX059 (MXB)
CK14	MX057 (MXB)	CD43	DF‐T1 (DAKO)
CK5/6	D5/16B4 (MXB)	TTF−1	8G7G3/1 (DAKO); MX011 (MXB)
P63	DAK‐P63(DAKO); MX013 (MXB)	Napsin A	MX015 (MXB)
S100	Multiclonal (DAKO)	K903	34βE12 (DAKO)
Calponin	CALP (DAKO)	CD10	56C6 (DAKO); MX002 (MXB)
SMA	1A4 (MXB)	E‐cadherin	NCH−38 (DAKO); MX020 (MXB)
Vimentin	Vim3B4 (DAKO); MX034 (MXB)	CD99	12E7 (DAKO); 013 (MXB)
SOX10	EP268 (MXB)	GFAP	Multiclonal
CEA	Ⅱ−7 (DAKO); Col−1 (MXB)	EGFR	SP111 (MXB)

All statistical analyses were performed using SPSS 22.0. To compare the distribution of the expression profiles of S100 proteins between the primary and metastatic cases, we performed the chi‐square test with Yates correction for the following reasons: the total number of cases (54 + 23+2 + 7 = 86) was more than 40, and the sample size in one of the groups (metastasis S100 (+), 2 cases) was greater than 1 but less than 5. Comparison of mean age according to tumour location (groups with<5 cases were excluded) was performed using one‐way analysis of variance (ANOVA) and LSD *t*‐test for two groups and more than two groups, respectively. All *p*‐values were two‐tailed. A *p*‐value < 0.05 was considered statistically significant.

## 3 RESULTS

**FIGURE 1:**
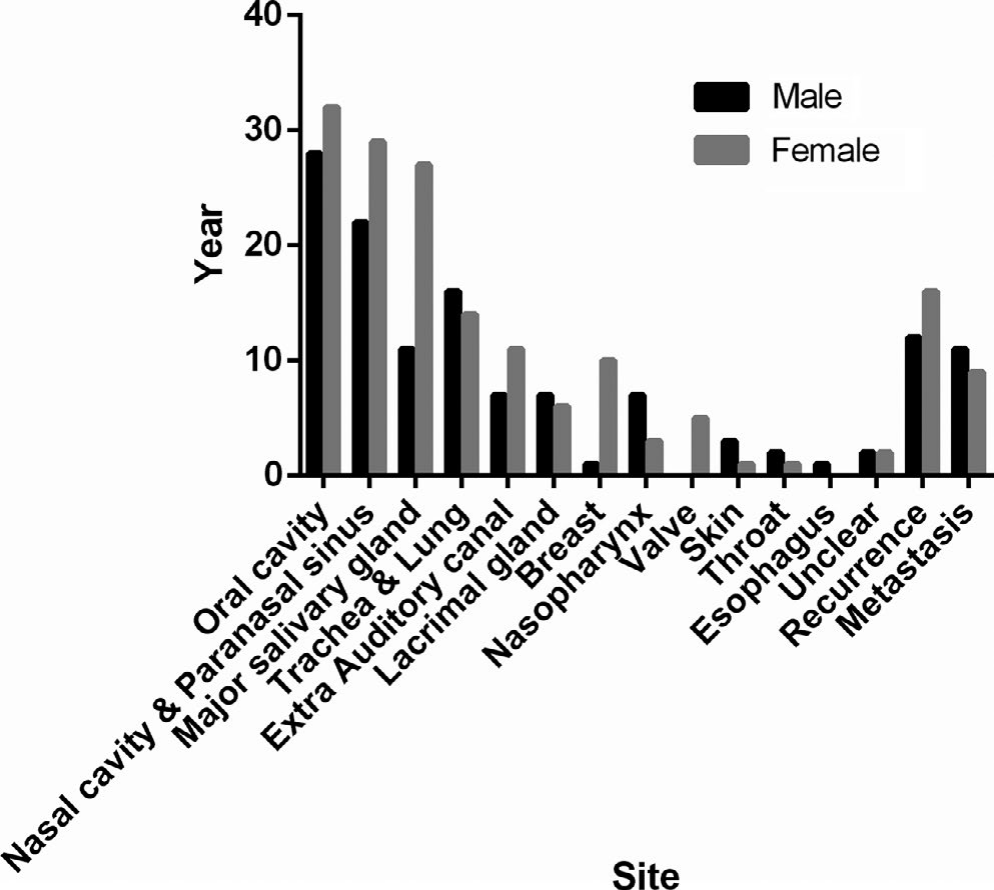
Sex ratio in each group

There were 248 primary cases (107 male and 141 female patients; ratio 1:1.31); a slight female predominance was seen (Figure 1). Fourteen cases were diagnosed as solid type ACC; the nasal cavity and paranasal sinuses group contributed the most with respect to this subtype (6/51, 11.7%). The number of margin positive cases was the highest in the oral cavity group (11/60). Perineural invasion was observed in 45 cases, most of which were a part of the major salivary gland (13/38, 34%) and oral cavity (10/60, 16.6%) groups. One case from the trachea and lung group involved vascular invasion. Synchronous LN metastasis was observed in two cases from the trachea and lung group (both cases: N1 stage disease). Twenty cases of metastasis, including two involving heterochronous LN metastasis, were observed after the primary surgery (Table 2).

**TABLE 2:** Analyses of 296 ACC cases according to sex, histological type, perineural invasion, vascular invasion, lymph node metastasis and high‐grade transformation

	Site	Case Number	Male sex	Female sex	Solid pattern	Margin positivity	Perineural invasion	Vascular invasion	LN positivity (stage)	High‐grade transformation
Primary (248)	Oral cavity	60	28	32	1	11	10	0	0	0
	Nasal cavity and paranasal sinuses	51	22	29	6	3	3	0	0	0
	Major salivary gland	38	11	27	2	2	13	0	0	0
	Trachea and lung	30	16	14	0	3	5	1	2 (N1)	1
	Extra auditory canal	18	7	11	0	0	2	0	0	0
	Lacrimal gland	13	7	6	0	1	3	0	0	0
	Breast	11	1	10	1	0	2	0	0	0
	Nasopharynx	10	7	3	0	0	0	0	0	0
	Vulva	5	0	5	0	2	1	0	0	0
	Throat	3	2	1	0	1	1	0	0	0
	Skin	4	3	1	0	1	0	0	0	0
	Unclear	4	2	2	1	0	1	0	0	0
	Oesophagus	1	1	0	1	0	0	0	0	0
Recurrence		28	12	16	1	2	4	0	0	0
Metastasis		20	11	9	1	0	0	0	2 (N1)	0
Total		296	130	166	14	26	45	1	4	1

Abbreviation: ACC, adenoid cystic carcinoma

**FIGURE 2:**
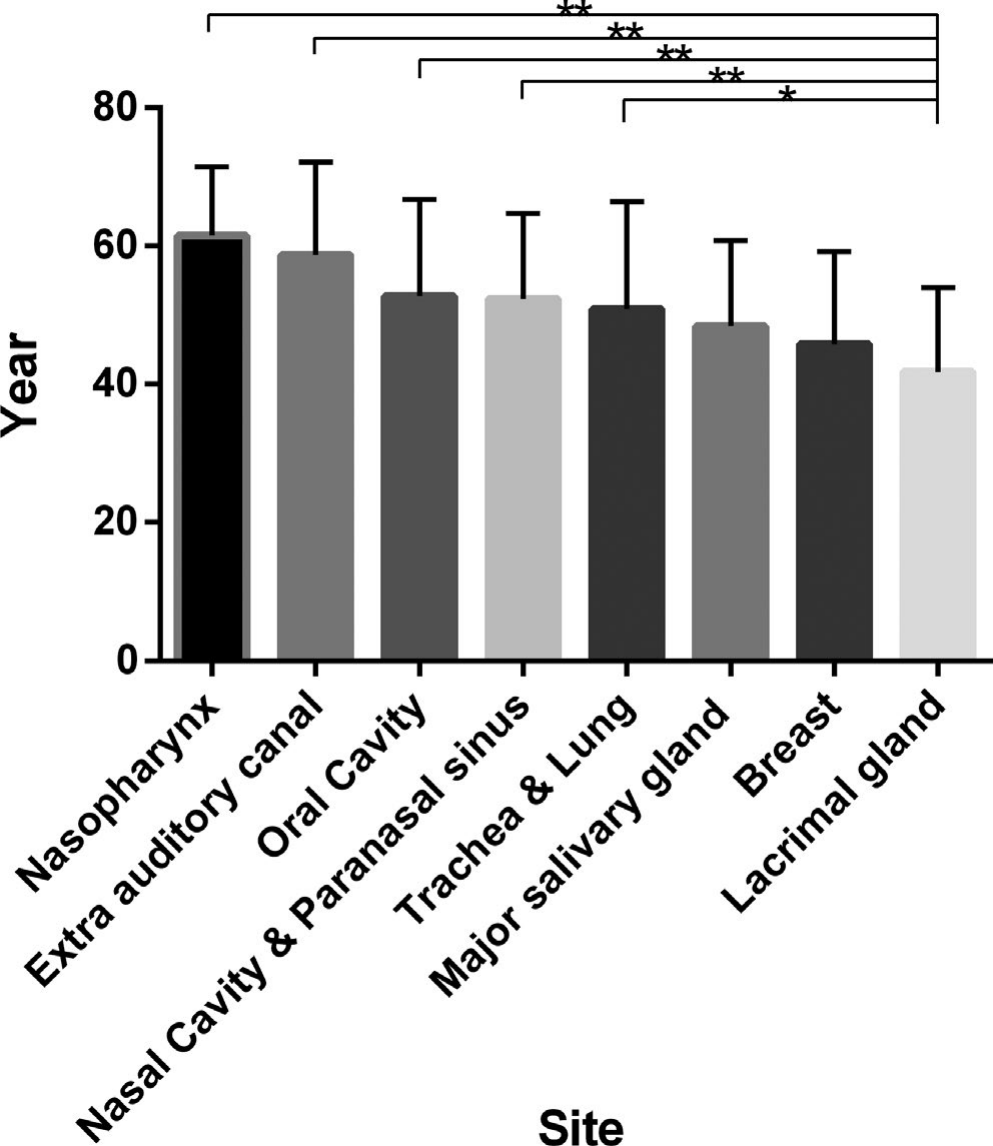
Mean age (standard deviation) in each group. Patients with adenoid cystic carcinoma arising from the lacrimal gland showed the lowest median age (39 years, mean = 41.85 years). The highest median age (60.5 years, mean = 59.7 years) was observed in the nasopharynx group. The detailed *p*‐values are presented in Table 3

The age of onset ranged from 12 to 87 years with a median age of 52 years. Patients with ACC arising from the lacrimal gland showed the lowest median age (39 years, mean =41.85 years). The median age of patients with ACC of the nasopharynx was 60.5 years (mean = 59.7 years) (Figure 2). Further, there were significant differences in mean age between the groups based on tumour site (Table 3, Figure 2).

**TABLE 3:** Analyses of mean age at onset according to tumour location （* *P*＜0.05; ** *P*＜0.01）

	Oral cavity	Nasal cavity and paranasal sinuses	Major salivary gland	Trachea and lung	Extra auditory canal	Lacrimal gland	Breast	Nasopharynx
Oral cavity	—	0.888	0.120	0.534	0.096	0.008**	0.113	0.055
Nasal cavity and paranasal sinuses	0.888	—	0.168	0.626	0.084	0.011*	0.138	0.049*
Major salivary gland	0.120	0.168	—	0.451	0.007**	0.124	0.564	0.006**
Trachea and lung	0.534	0.626	0.451	—	0.050	0.041*	0.279	0.030*
Extra auditory canal	0.096	0.084	0.007**	0.050	—	0.001**	0.012*	0.597
Lacrimal gland	0.008**	0.011*	0.124	0.041*	0.001**	—	0.467	0.001**
Breast	0.113	0.138	0.564	0.279	0.012*	0.467	—	0.007**
Nasopharynx	0.055	0.049*	0.006**	0.030*	0.597	0.001**	0.007**	—

**FIGURE 3:**
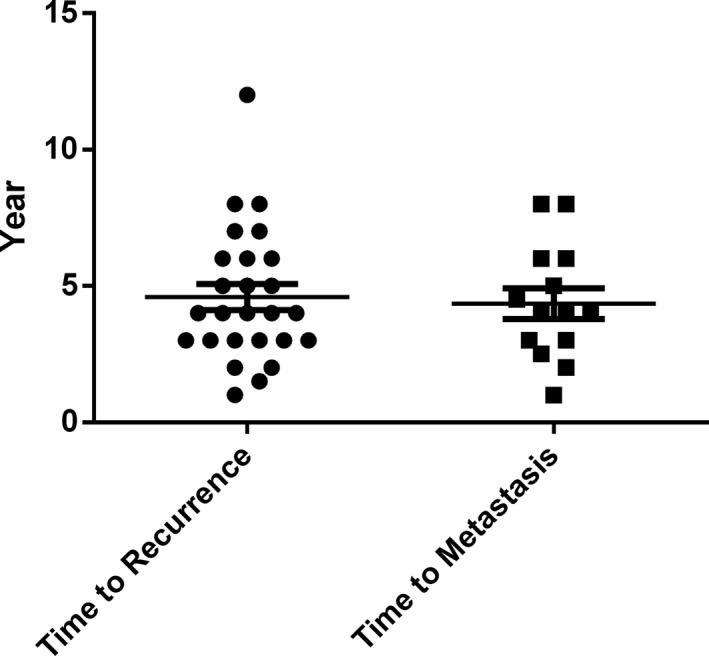
Scatter diagram of the time to recurrence or metastasis after surgery

The period between surgery and tumour recurrence ranged from 1 to 12 years, with an average of 4.95 years. Metastasis occurred 1–8 years after surgery (mean = 4.35 years) (Figure 3).

Duration between the onset of symptoms and diagnosis ranged from 1 week to 360 months, with the mean of 19.8 months in 118 patients with traceable medical records. The shortest average duration (5.3 months) between the onset of symptoms and diagnosis was observed in the nasopharynx group and the longest mean duration (88 months) was observed in the skin group.

Eighty‐six patients presented with intact tumour masses, with sizes ranging from 0.7 to 8 cm. Preoperative imaging reports were available for nine patients, based on which the size of the tumour was measured (range: from 0.37 to 6.3 cm). The average size of the tumour was 2.7 cm.

**FIGURE 4:**
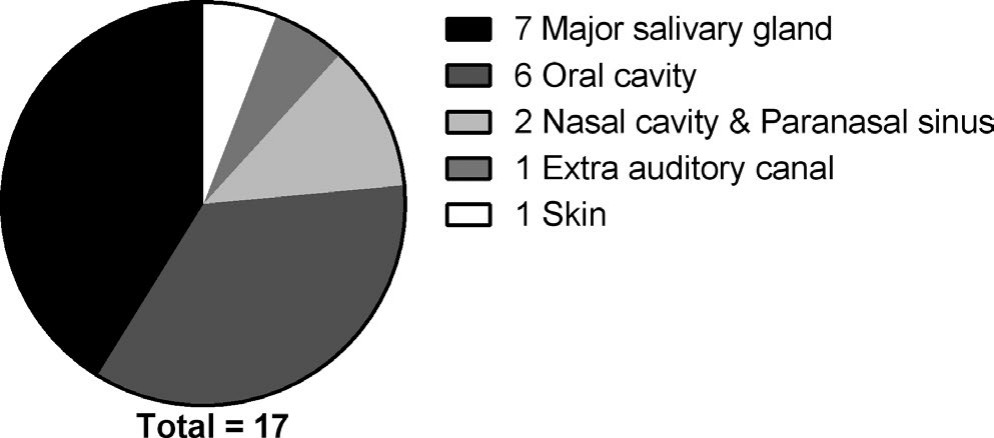
Primary sites of tumours that showed metastasis

**FIGURE 5:**
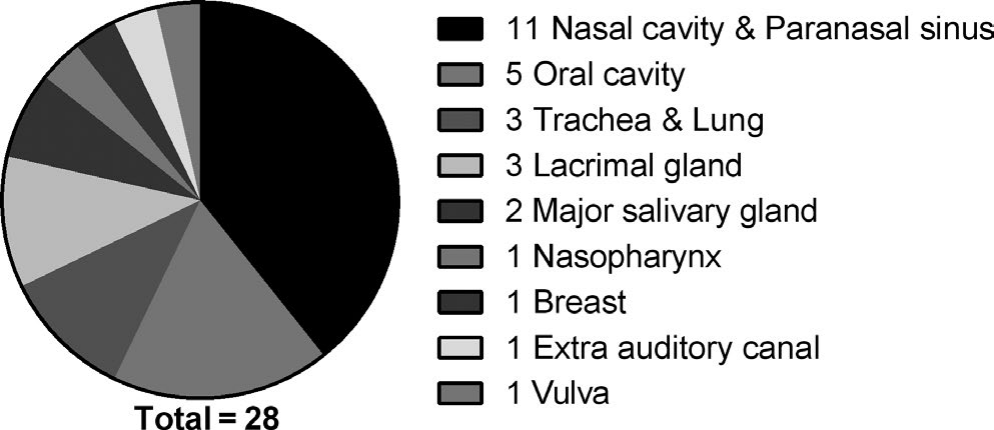
Summary of the recurrence sites

The most and least vulnerable sites were the oral cavity (n = 60) and oesophagus (n = 1), respectively (Table 2). Tumours arising in the major salivary glands and the mouth cavity were more likely to metastasise to distant sites than tumours arising in other locations (Figure 4). Interestingly, tumours arising from the nasal cavity and paranasal sinuses showed local recurrence (Figure 5).

The lung was the most common site of metastasis (n = 14), followed by the liver (n = 3), lymph nodes (n = 2), bone (n = 1) and chest wall (n = 1); in one case, metastases to both the lungs and bone were seen. Contrary to most carcinomas, ACC metastasises more commonly to the lung than to regional lymph nodes.

Immunohistochemistry was performed in 135 cases. The results are shown in Table 4. S100 proteins were expressed in 54 primary and 2 metastatic cases but were not expressed in 23 primary and 7 metastatic cases (*p* = 0.013).

**TABLE 4:** Immunohistochemistry results

Antibody	Number of positive cases	Antibody	Number of positive cases
PCK	56/56 (100%)	CK19	3/3 (100%)
CK7	87/88 (98.8%)	CD56	8/12 (66.6%)
EMA	42/43 (97.6%)	P40	13/16 (81.2%)
CD117	113/120 (94.1%)	Bcl−2	14/14 (100%)
CK8/18	35/35 (100%)	SMMHC	12/14 (85.7%)
CAM5.2	2/2 (100%)	CK20	1/6 (16.6%)
CK14	26/27 (96.2%)	CD43	3/11 (27.2%)
CK5/6	73/73 (100%)	TTF−1	5/24 (20.8%)
P63	111/124 (89.5%)	Napsin A	1/5 (20.0%)
S100	61/92 (66.3%)	K903	1/1 (100%)
Calponin	41/50 (82.0%)	CD10	0/8 (0.0%)
SMA	38/43 (88.3%)	E‐cadherin	3/3 (100%)
Vimentin	32/32 (100%)	CD99	0/2 (0.0%)
SOX10	36/36 (100%)	GFAP	2/22 (9.0%)
CEA	23/34 (67.6%)	EGFR	3/4 (75.0%)

Abbreviations: CEA, carcinoembryonic antigen; EGFR, epidermal growth factor receptor; EMA, epithelial membrane antigen; GFAP, glial fibrillary acidic protein; SMA, smooth muscle actin; SMMHC, smooth muscle myosin heavy chain; TTF‐1, thyroid transcription factor‐1.

## 4 DISCUSSION

ACC commonly shows a female predominance. This might be attributed to a unique mechanism involving oestrogen. A previous study demonstrated that the oestrogen receptor (ER) is expressed in the salivary glands.^11^, ^12^ Upregulation of ER enhances the malignant phenotype of the ACC cell line that normally does not express this receptor.^13^ Tamoxifen, an ER antagonist, has been effective in the clinical control of ACC.^14^, ^15^, ^16^ We observed that patients with ACC of the lacrimal glands were younger than those with tumours at other sites of the body. This might be due to the unique microRNA signature of ACC of the lacrimal gland, although tumours share the same morphology and a similar genetic landscape irrespective of the site of origin.^10^ In contrast to our results, a much higher median age (54 years) was reported in one study involving 24 cases of lacrimal gland ACC.^17^ This might be attributed to the difference in sample size. In contrast, ACC of the extra auditory canal and nasopharynx commonly affects elderly individuals; however, the underlying mechanism remains unclear. Some researchers have unsuccessfully attempted to explain the relationship between nasopharynx ACC and infection with Epstein–Barr virus.^18^

ACC of the major salivary glands, especially the submandibular gland, was chiefly associated with distant metastasis, although they were less prevalent than ACC of the nasal cavity and paranasal sinuses.^19^, ^20^, ^21^ The high expression of *MYB* mRNA might explain the high frequency of metastasis from tumours of the major salivary gland, especially those of the submandibular gland.^22^, ^23^

In our study, 54 (54/77) primary cases showed positivity for S100, but seven of nine metastatic cases showed negativity for S100 (7/9) (*p* = 0.013). A plausible reason for the lack of S100 expression is the absence of myoepithelial cells or the lack of expression of S100 proteins despite the presence of myoepithelial cells. In our cases, S100 negativity was not a result of the absence of myoepithelial cells since positive results were obtained for several other markers of these cells, such as CK5/6, P63 and calponin. The loss of S100 expression was probably due to the loss of function of myoepithelial cells in metastatic ACC.^24^ Cancers comprising myoepithelial cells commonly have a low grade and have the ability to secrete several extracellular matrix proteins.^4^ Evidence suggests that myoepithelial cells are tumour inhibitors as they can suppress cell division and invasiveness by secreting factors that induce the suppression of epithelial cell division and invasiveness, myxoid substances and basement membrane components.^25^, ^26^ A breast cancer study reported decreased expression of laminin‐1 in tumour‐associated myoepithelial cells, suggesting a strong negative correlation between laminin‐1 and cancer.^27^ S100 proteins, a family of Ca^2+^‐binding proteins, are involved in regulating several cellular functions.^28^ Loss of the expression of S100 proteins in myoepithelial cells appears to be essential for the metastasis of tumour cells to remote sites. A study reported that S100 proteins could bind to specific sites on PC12 cells and induce apoptosis.^29^ *In vivo* and *in vitro* experiments have shown that at low concentrations, the extracellular S100‐beta protein acts as a growth differentiation factor in neuronal and glial cells.^6^ Although the four myoepithelial markers P63, SMA, S100 and calponin can be used to prove the presence of myoepithelial cells, based on our limited data from nine metastatic cases, S100 might be a good prognostic marker for predicting distant metastasis from ACC. A larger sample size is needed for further evaluation of the prognostic role of S100 in ACC metastasis. The absence of multiple markers might be correlated with a poor prognosis.

We observed that ACC of the nasal cavity and paranasal sinuses is likely to be correlated with local recurrence. Although the oral cavity group contributed the most with respect to cases involving margin positivity (11/60) and perineural invasion (10/60) (Table 2), the recurrence rate of tumours of the nasal cavity and paranasal sinuses (11/28) was twice as that of tumours of the oral cavity (5/28) (Figure 5). One explanation for this result could involve the sophisticated anatomy of the nasal cavity and paranasal sinuses, which facilitates the invasion of the tumour into surrounding regions (oral cavity, soft tissues, pterygomaxillary fossa, orbit and skull base) and aids in tumour extension along the branches of the pterygopalatine nerve. Another reason could be that the samples from the nasal cavity and paranasal sinuses were fragmented, making it difficult to identify positive margins.

To the best of our knowledge, this is the largest case study on ACC. Further, this study is the first to evaluate the prognostic role of the loss of S100 expression in ACC metastasis.

The study had some limitations. It would have been better if the number of metastatic cases was higher. Another limitation was the absence of genetic analyses regarding the relation between the absence of S100 expression and metastasis, which will be our focus in further studies on this topic.

## FINANCIAL DISCLOSURE

None declared.

## CONFLICTS OF INTEREST

All authors have no conflicts of interest.

## AUTHOR CONTRIBUTION

Guarantor of integrity of entire study Changzheng Huang; Study concepts Shidi Wu; Study design Xiu Nie; Literature research Hongfeng Zhang; experimental studies Zheng Huang; Data acquisition Juan Pan; Data analysis/interpretation Ting Wu, Haihua Ye; Statistical analysis Jiaorong Chen; Manuscript preparation Zheng Huang.

## Data Availability

Some or all data, models or code generated or used during the study are available from the corresponding author by request. (List items). The data generated and analysed during research are included in this article.
